# Effects of 4-Week Intensive Active-Resistive Training with an EMG-Based Exoskeleton Robot on Muscle Strength in Older People: A Pilot Study

**DOI:** 10.1155/2016/1256958

**Published:** 2016-02-23

**Authors:** Jongsang Son, Jeseong Ryu, Soonjae Ahn, Eun Joo Kim, Jung Ah Lee, Youngho Kim

**Affiliations:** ^1^Sensory Motor Performance Program, Rehabilitation Institute of Chicago, Chicago, IL 60611, USA; ^2^Department of Physical Medicine and Rehabilitation, Northwestern University, Chicago, IL 60611, USA; ^3^Department of Biomedical Engineering, Yonsei University, Wonju, Gangwon 220-710, Republic of Korea; ^4^Department of Rehabilitation Medicine, Korea National Rehabilitation Center, Seoul 142-884, Republic of Korea; ^5^Department of Clinical Research on Rehabilitation, Korea National Rehabilitation Research Institute, Seoul 142-884, Republic of Korea

## Abstract

This study aims to investigate the idea that an active-resistive training with an EMG-based exoskeleton robot could be beneficial to muscle strength and antagonist muscle cocontraction control after 4-week intensive elbow flexion/extension training. Three older people over 65 years participated the training for an hour per session and completed total 20 sessions during four weeks. Outcome measures were chosen as the maximum joint torque and cocontraction ratio between the biceps/triceps brachii muscles at pre-/post-training. The Wilcoxon signed-ranks test was performed to evaluate paired difference for the outcome measures. As a result, there was no significant difference in the maximum flexion or extension torque at pre- and post-training. However, the cocontraction ratio of the triceps brachii muscle as the antagonist was significantly decreased by 9.8% after the 4-week intensive training. The active-resistive training with the exoskeleton robot in the older people yielded a promising result, showing significant changes in the antagonist muscle cocontraction.

## 1. Introduction

Aging is associated with a loss of muscle mass (i.e., sarcopenia), so that the functional muscle strength would be diminished [1]. Such muscle weakness is considered the primary cause of disability for most people with neuromuscular diseases and injuries to the central nervous system [2], which eventually might limit activities of daily living. A number of previous literatures regarding the loss of static or dynamic strength implied that the functional muscle strength is typically declined by 30–40% over the total span of adult life. Interestingly, the strength would be maintained to about 45 years of age but deteriorated rapidly afterwards, so that there is a 25% functional impairment by the age of 65 years [3]. This evidence strongly supports that strength training for older people is recommended to increase muscle strength and, ultimately, to gain independence in activities of daily living [4, 5].

Knowing an individual's weight capacity is important to establish an exercise protocol in strength training [6], and the one-repetition maximum (1-RM) which is the greatest amount of weight that a person is able to lift in a single repetition is widely used for determining an individual's maximum strength. However, 1-RM might not be appropriate for older people unaccustomed to weight training due to an increased risk of muscle soreness and serious musculoskeletal injury from lifting maximal weight levels [7].

Robotic devices have arisen as an assistance to trainers or therapists, providing safe, high-intensive, task-specific, and repetitive trainings or rehabilitation, and also can be used as more objective and reliable evaluation tools [8, 9]. Typical protocol with such devices is based on a passive or an active training. Contrary to the passive training with less volition, an individual's voluntary efforts could be induced in the active training. According to some literatures [10, 11], the passive training technique such as continuous passive movement showed less advantages in terms of functional improvements. Therefore, most studies have been performed to utilize the combined technique such as the active-assistive or active-resistive training for the purpose of functional improvements and demonstrated that the active-related training is better for improvements of motor functionality than the passive training [12]. In this regard, our group has developed an EMG-based exoskeleton robot for the active-resistive training of biceps and triceps brachii muscles [13].

To the best of our knowledge, a number of studies have used robotic devices for a population of patients with neuromuscular disorders such as stroke [8–12, 14–16] and spinal cord injury [17–20]; that is, little research has been performed to show feasibility of robotic training for older people. Since robotic devices provide an easily adjustable resistance with no physical weights, it was hypothesized that an active-resistive training with a robotic device would become an alternative of conventional strength training that might not be appropriate for older people. To investigate the hypothesis, this pilot study aims at the idea that an active-resistive training with an EMG-based exoskeleton robot could be beneficial to muscle strength and antagonist muscle cocontraction control in older people after 4-week intensive training.

## 2. Methods

### 2.1. Subjects

Three elderly people (age: 69.0 ± 3.6 yrs.; height: 164.7 ± 2.9 cm; and weight: 65.3 ± 7.8 kg) participated, who gave the informed consent approved by the Institutional Review Board of the National Rehabilitation Center. Inclusion criteria were being over 65 years of age and normal abilities of visual perception and recognition. People with an arthritis or other clinical opinions were excluded.

### 2.2. Evaluation Procedure

**Figure 1:**
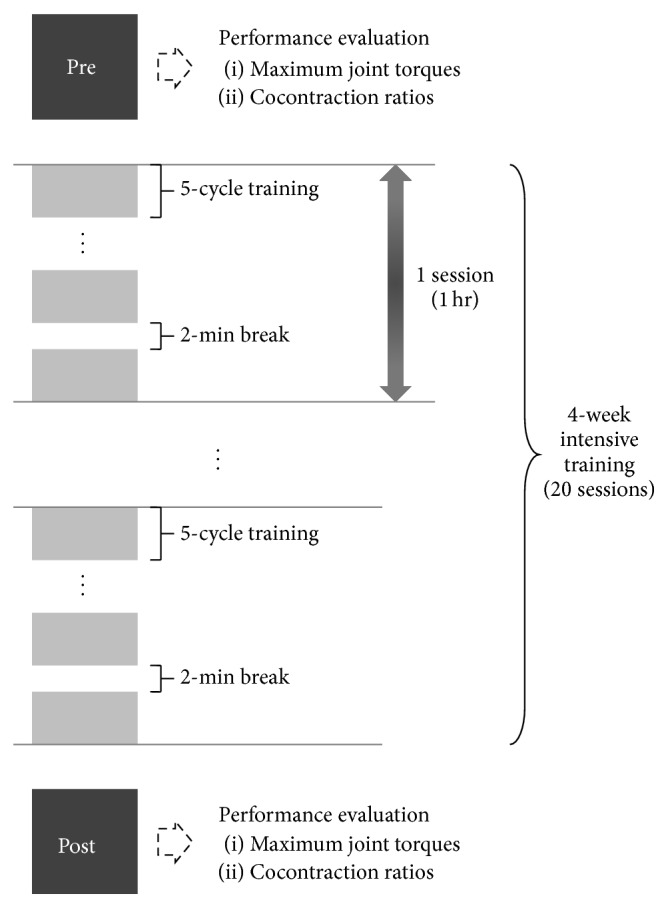
Experimental sessions during 4-week intensive training.

The subjects trained for an hour per session and completed total 20 sessions during four weeks. A session consists of multiple trials and 2-minute breaks between each trial that is defined as 5 cycles of repetitive elbow flexion and extension (Figure 1). The number of repeated trials in a session depended on subjects, but it was 12–14 on average. At pre-/post-training, the participant was asked to perform three maximum voluntary isometric contractions (MVIC) for the muscle group of interest (i.e., biceps and triceps brachii muscles), separated by a 30-second rest, with an elbow joint angle of 90° on an isokinetic exercise device (Biodex System 3 Pro, Biodex Medical Systems, USA) synchronized with an EMG measurement system (Trigno Wireless Systems, Delsys Inc., USA). The elbow joint torque and EMG signals from two muscles were measured at 1 kHz. Outcome measures were chosen as the maximum joint torque and cocontraction ratio (CR) between the biceps/triceps brachii muscles and were determined using the 10-second contraction data from three MVIC trials. The cocontraction ratio was defined as follows:

(1) $$\begin{array}{c} \text{CR}=\frac{\int_{t_{1}}^{t_{2}}{\text{EMG}}_{\text{ant}}dt}{\int_{t_{1}}^{t_{2}}\left({\text{EMG}}_{\text{ago}}+{\text{EMG}}_{\text{ant}}\right)dt}\times 100, \end{array}$$

where *t*_1_ is the movement onset, *t*_2_ the movement end, EMG_ago_ the linear-enveloped EMG of the agonist pair, EMG_ant_ the linear-enveloped EMG of the antagonist pair, and *dt* the time interval (i.e., 0.001 s).

### 2.3. EMG-Based Exoskeleton Robot

**Figure 2:**
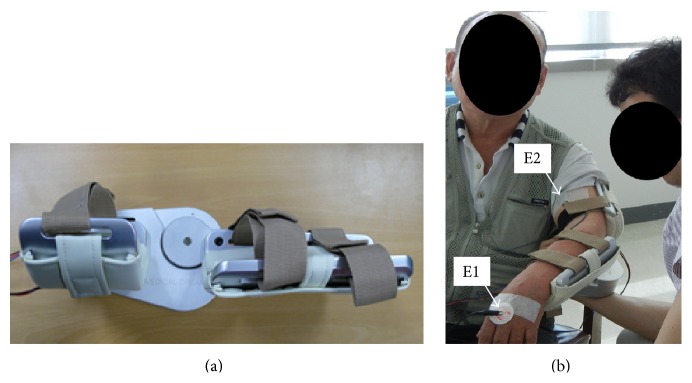
The developed EMG-based active-resistive training system (a) and its experiment (b). E1 indicates the ground electrode and E2 the EMG sensor for biceps brachii muscle. EMG sensor for triceps brachii muscle was not shown due to the exoskeleton.

EMG-based exoskeleton robot for the active-resistive training of elbow muscles is an advanced version of our previous system [13]. In brief, the system includes a brushed DC motor (GR 53 × 30, Ametek PMC, US) to generate elbow flexion and extension movements, with a constant velocity of 10°/s, and an exoskeleton to fix the arm. The exoskeleton was designed to allow the elbow joint range of motion from 0° (fully extended) to 140° (fully flexed). Two custom-made EMG sensors (bandwidth: 20–450 Hz) and one ground electrode were attached on biceps/triceps brachii muscles and metacarpal head bone with a medical adhesive tape, respectively (Figure 2). The raw EMG signals sampled at 1 kHz were high-pass filtered using a fourth-order Butterworth filter (20 Hz) to remove motion artifact, full-wave rectified, and then low-pass filtered using the same filter with 6 Hz cut-off frequency. The linear-enveloped signals were then normalized using values obtained during maximal voluntary isometric contraction. Such muscle activities were used to control the elbow joint since a simple EMG-triggered on-off control has been used to apply the EMG-controlled robot-aided therapy for people after stroke [21]. The simple EMG-triggered on-off control-based exercise algorithm is that the joint movement is allowed only when the muscle activity is greater than the preset threshold ranged between 0 and 1. For example, if the preset threshold value for the biceps brachii muscle is 0.5, the elbow joint would be allowed to flex only when the muscle activity is greater than 0.5.

### 2.4. Statistical Analysis

Statistical analyses were performed using IBM SPSS Statistics (Version 20, IBM, Armonk, USA). Based on the significance level of 0.05, the Kolmogorov-Smirnov and the Levene tests were performed to assess assumptions of the normality and homogeneity of variances, respectively. Since all data did not satisfy the assumptions, the Wilcoxon signed-ranks test was performed to evaluate paired difference of the outcome measures. Moreover, the data were presented as median (i.e., the value that is the middle of the distribution) and interquartile range (i.e., the range of values within reside the middle 50% of the distribution, IQR). The lower bound of IQR is called the first quartile (Q1) and the upper bound the third quartile (Q3). Thus, IQR was determined as Q1–Q3.

## 3. Results

**Figure 3:**
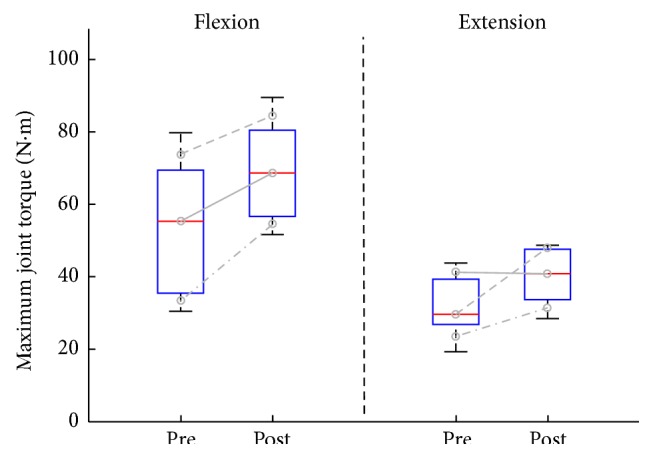
Changes in maximum joint torques between pre-training (Pre) and post-training (Post). Boxes represent the interquartile range (25th to 75th percentile), lines in the boxes the median (50th percentile), and whiskers the 5th and 95th percentiles. Three lines in each side indicate changes in the maximum joint torque of an individual.

Figure 3 shows the maximum joint torque at pre-/post-training. At pre-training, the median of the maximum flexion torque was 55.2 N·m (IQR = 35.3–69.3), and the median of the maximum extension torque was 29.5 N·m (IQR = 26.7–39.2). After the 4-week intensive training, the median maximum torque was increased by 68.5 N·m (IQR = 56.5–80.4) for the flexion and 40.7 N·m (IQR = 33.5–47.5) for the extension. There was no significant difference in both maximum joint torques.

**Figure 4:**
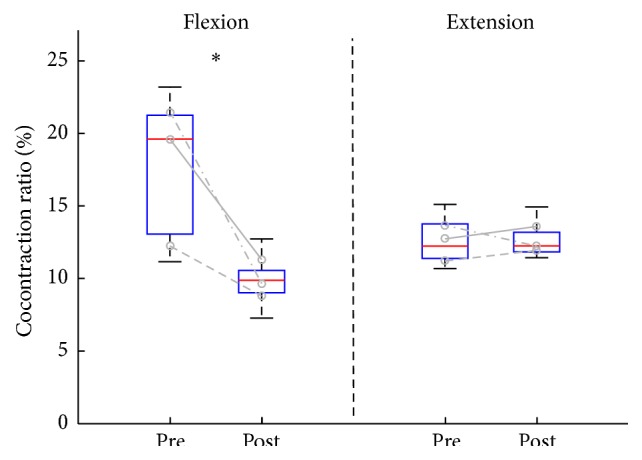
Changes in cocontraction ratios between pre-training (Pre) and post-training (Post). Boxes represent the interquartile range (25th to 75th percentile), lines in the boxes the median (50th percentile), and whiskers the 5th and 95th percentiles. Three lines in each side indicate changes in the cocontraction ratio of an individual. An asterisk indicates significant difference between Pre and Post.

Figure 4 shows changes in the cocontraction ratio. Before the training, the median of the cocontraction ratio of the triceps brachii muscle as the antagonist was 19.6% (IQR = 13.0–21.2) but significantly decreased by 9.8% (IQR = 9.0–10.5) (*p* < 0.001) after the training. In case of the biceps brachii muscle as the antagonist, there was no significant change showing the median of 12.2 (IQR = 11.4–13.7) at pre-training and 12.2 (IQR = 11.8–13.2) at post-training.

## 4. Discussion

To the best of our knowledge, most studies with the robotic devices have been performed for patients with neuromuscular disorders. However, older people also obviously need a proper strength training to defer diseases with the natural aging. In this regard, this study dealt with the effects of an active-resistive training with the EMG-based exoskeleton robot on muscle strength and cocontraction of the older people. To evaluate the changes in the muscle performance after 4-week intensive training, the maximum joint torque and cocontraction ratio were chosen as outcome measures. The maximum joint torques did not change, but the cocontraction ratio of triceps brachii muscles did significantly reduce by 9.8% during the elbow flexion. This implies that an active-resistive training with a robotic device could be a promising strength training for older people by providing a relatively safer resistance load than a physical weight.

The decrease in muscle strength can be partly explained by the decreased activation of the agonist muscles and/or changes in the degree of the agonist-antagonist cocontraction ratio [22]. The greater degree of the antagonist muscle cocontraction was observed in the older people compared with the young people [23]. This study did not compare the changes in the cocontraction ratio between the young and older people, because the purpose of this study was to investigate effects of an active-resistive training with a robotic device on the muscle performance of the older people, not to compare the characteristics in the young and older people. Thus, we could not prove whether functionalities of the participants in this study were different compared with the young people or not. However, our results showed that the cocontraction ratio significantly decreased by 9.8% in case of the triceps brachii muscle as the antagonist, which could explain the improvements in the maximum joint torques though there was no significance. Hence, we suggest that at least an EMG-based active-resistive training was good for improvements in ability to control the antagonist muscle cocontraction after 4-week intensive training.

The effects of robot-mediated training on the muscle cocontraction have been reported in studies with neuromuscular patients. Hu et al. [14] investigated the variation of muscle coactivation patterns according to the robot-assisted rehabilitation (i.e., active-resistive training by tracking a target cursor moving on the screen) on elbow flexion and extension for chronic stroke during the 7-week training. As a result, the cocontraction between biceps and triceps brachii muscles significantly decreased after the training period, and this decrease was related to the improvement in the tracking accuracy (i.e., the coordination of the individual muscles). Posteraro et al. [15] also compared the effects of two robot-mediated therapies on spasticity in patients with chronic hemiparesis and concluded that the active movement training was not responsible for an increase in spasticity but results in the reduced hypertonia (i.e., spasticity reduction) in antagonist muscles by activating the reciprocal inhibition mechanism. Unfortunately, since these results were obtained from the neuromuscular patients, we could not directly connect the interpretation of the previous results with our results. In addition, the muscle cocontraction is considered to control the joint stability in the static condition [24] and to facilitate multijoint arm movement accuracy in the static condition [25]. However, it might be corresponding to the people with no functional impairment [14]. Indeed, excessive cocontractions need high energetic cost [25] and might cause the attenuated force production in the elderly people [23]. Thus, we believe that the decreased cocontraction in the elderly people might be acceptable as a possible mechanism of improvements in muscle strength from an intensive robotic active-resistive training.

Our findings are limited to be generalized due to the small number of subjects. It is a potential reason for no significant difference in the maximum joint torques, although the average values slightly increased after the 4-week training. This might lead a need of further experiments to confirm a similar consistency with the current results. In addition, one simple strength training protocol was applied in this study even though the current study showed that an active-resistive training with a robotic device could be a promising strength training for elderly people. Indeed, a protocol for muscle strength training has been developed and has proven effects in various ways [26, 27]. Future studies of how numerous protocols of conventional strength training can be linked with a robotic device would be expected.

## 5. Conclusion

In summary, this study showed that the 4-week intensive active-resistive training with the exoskeleton robot results in the decreased cocontraction in the elderly people. We believe that this study would be valuable for further studies with robotic devices for elderly people, showing significant changes in the antagonist muscle cocontraction.
